# Dimerization of Hepatitis E Virus Capsid Protein E2s Domain Is Essential for Virus–Host Interaction

**DOI:** 10.1371/journal.ppat.1000537

**Published:** 2009-08-07

**Authors:** Shaowei Li, Xuhua Tang, J. Seetharaman, Chunyan Yang, Ying Gu, Jun Zhang, Hailian Du, J. Wai Kuo Shih, Choy-Leong Hew, J. Sivaraman, Ningshao Xia

**Affiliations:** 1 National Institute of Diagnostics and Vaccine Development in Infectious Disease, School of Life Sciences, Xiamen University, Xiamen, China; 2 Xiamen-NUS Joint Laboratory in Biomedical Sciences, Xiamen University, Xiamen, China; 3 Department of Biological Sciences, National University of Singapore, Singapore; 4 X4 Beamline, Brookhaven National Laboratory, Upton, New York, United States of America; Institut Pasteur, France

## Abstract

Hepatitis E virus (HEV), a non-enveloped, positive-stranded RNA virus, is transmitted in a faecal-oral manner, and causes acute liver diseases in humans. The HEV capsid is made up of capsomeres consisting of homodimers of a single structural capsid protein forming the virus shell. These dimers are believed to protrude from the viral surface and to interact with host cells to initiate infection. To date, no structural information is available for any of the HEV proteins. Here, we report for the first time the crystal structure of the HEV capsid protein domain E2s, a protruding domain, together with functional studies to illustrate that this domain forms a tight homodimer and that this dimerization is essential for HEV–host interactions. In addition, we also show that the neutralizing antibody recognition site of HEV is located on the E2s domain. Our study will aid in the development of vaccines and, subsequently, specific inhibitors for HEV.

## Author Summary

Infectious viral hepatitis is a major disease in both developing and developed countries. Hepatitis E virus (HEV) is one of the major causes of severe inflammation of the liver, which is characterized by jaundice, fever, liver enlargement, and abdominal pain in humans and non-human primates. The hepatitis E virus capsid is made up of individual subunits consisting of homodimers of a single structural protein forming the virus shell. These dimers are believed to protrude from the viral surface and to interact with host cells to initiate infection. To date, no structural information is available for any of the HEV proteins. This article reports the crystal structure of the HEV capsid protein domain E2s (protruding domain), along with functional studies, which illustrate the tight homodimeric state of E2s and that dimerization is essential for both HEV–host interactions and disease progression. We also show that the neutralizing antibody recognition site of HEV is located on the E2s domain. The present findings will aid the development of vaccines and novel inhibitors for HEV.

## Introduction

Infectious viral hepatitis is a major health problem in both developing and developed countries. Hepatitis E virus (HEV) is an important cause of severe hepatitis in humans and is responsible for unusually high rates of mortality in pregnant women by the development of fulminant liver disease [1]. HEV morphologically resembles the *Calicivirus* and had been initially classified into the family of *Caliciviridae*. However, sequence comparisons and phylogenetic taxonomy differentiate HEV from *Calicivirus*, and it now defines a new family named *Hepeviridae* [2],[3]. This family has at least four recognized genotypes, but with a single serotype [4]. The HEV genome is a positive-stranded RNA of approximately 7.5 kb that encodes at least three different proteins. One of these genes (ORF2) encodes a single structural protein (pORF2) of 660aa. A 22 Å low- resolution cryoEM structure of recombinant HEV virus-like particles shows that the virus capsid is made up of subunits (capsomeres) consisting of homodimers of this structural protein [5]. Subunits of this dimeric capsid protein interact through their dimeric C-terminal domain to form a virus shell that protrudes from the viral surface [5],[6]. The initial contact with host cells to initiate viral infection is believed to occur through these protrusions [7].

Our previous studies on a number of recombinant HEV viral capsomeres derived from E2 protein (aa394–606) suggested that the dimeric domain encompasses aa459–606, of which, aa597–602 are involved in dimer formation. In addition, regions spanning aa394–459 and aa607–660 are believed to be involved in the stabilization of the homodimers [6]. Monoclonal antibodies reactive against the abovementioned regions bind to live HEV, and at least two monoclonal antibodies (8C11 and 8H3) could neutralize the infectivity of HEV [8]. A recombinant mutant of E2, p239 (ORF2 aa368–606), forms particles of diameter 23 nm, presumably via dimeric interactions [6],[9]. These particles could specifically adsorb and penetrate susceptible host cells similarly to live viruses. This interaction could be blocked by the neutralizing monoclonal antibodies mAb 8C11 and 8H3 [7], thus suggesting that the dimeric domain of these polypeptides resembles the virus capsid (capsomeres including the neutralization sites), most probably the surface protrusion.

As a continuation of our efforts to understand the structure and function of the Hepatitis E virus and its proteins, here we report the crystal structure of the dimerization domain of the recombinant capsid protein E2 (hereafter referred to as E2s, located on ORF2 aa455–602) refined up to 2.0 Å resolution. This is the first report of a crystal structure of a HEV protein. E2s has a β-barrel architecture consisting of an internal hydrophobic pore with both sides of the β-barrel blocked by short loops. The structure-based site-directed mutagenesis targeting the dimer interface, as well as the surface groove of the E2s domain, i.e. the proposed neutralizing antibody binding site, showed that the E2s domain is lying in the region of HEV that is likely to be involved in host interactions for effective propagation of viral infection. Further, our studies suggest that the dimerization of capsid E2s domain is a prerequisite for the virus-host interaction as well as for the binding of some neutralizing antibodies to HEV.

## Results

### Overall structure

**Figure 1:**
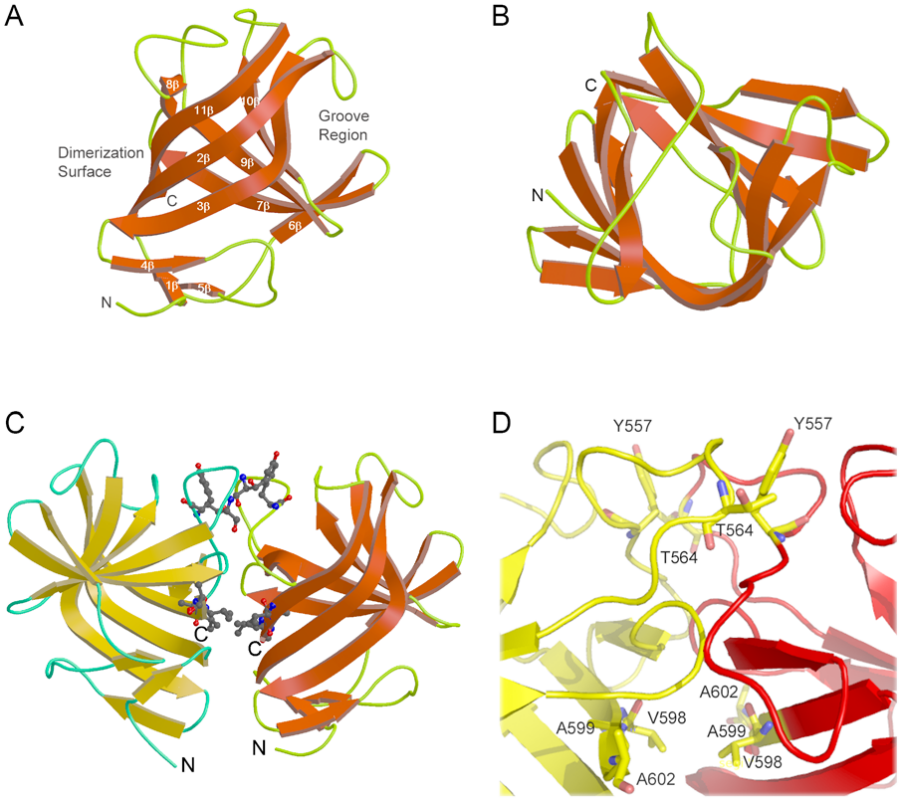
Structure of E2s. *(A)* Ribbon diagram of the subunit of the E2s dimer, side view. *(B)* Top view of the subunit of E2s dimer showing the cavity. β-strands and random coils/turns are depicted in red and green respectively. N- and C-termini are labeled. The dimerization interface and groove region are labeled. *(C)* The E2s dimer. Subunit A is shown in yellow, subunit B in red. Dimeric interface residues from both subunits are shown in ball-and-stick representation. Notably, the asymmetric unit consists of one subunit of the dimer. This dimer is generated by crystallographic symmetry. These figures were prepared by using Molscript and Raster3D [32],[33]. *(D)* Close-up view of the dimer interface. Key residues involved in the dimerization are labeled. This figure was prepared using PyMol [34].

The structure of recombinant E2s domain from Hepatitis E Virus (HEV) capsid was solved by the Single-wavelength Anomalous Dispersion (SAD) method from a synchrotron data set using Br heavy atom soaked crystals. The model was refined to a final R-factor of 0.198 (R_free_ = 0.240) at 2.0 Å resolution (Table 1). The E2s model consists of residues from Ser459 to Ala602. Five residues at the N-terminus had no interpretable electron density map and were not modeled. The asymmetric unit consists of one E2s molecule (Figure 1A and 1B). Notably, the symmetry related molecules maintain the tight dimeric architecture of E2s (Figure 1C and 1D).

**Table 1:** Data Collection and Refinement Statistics.

*Category*		*Data Set*
***Data Collection***	Cell parameters (Å, °)	a = b = 111.45, c = 84.33
		α = β = 90, γ = 120
	Space group	R32
	Resolution range (Å)	50−2.0 (2.07−2.0)
	Reflections (total/unique)	141359/24473
	Redundancy	5.8 (3.8)
	Completeness (%)	92.6 (68.2)
	R_sym_ a	0.088 (0.269)
***Refinement***	Resolution range (Å)	25.0−2.0
	R_work_ b (Number of Reflections)	0.1975 (20153)
	R_free_ c (Number of Reflections)	0.2401 (1006)
	RMSD bond lengths (Å)	0.006
	RMSD bond angles (°)	1.400
***Ramachandran Plot***	Most favored region (%)	80.2
	Additional allowed regions (%)	17.5
	Generously allowed regions (%)	2.4
	Disallowed regions (%)	0.0

Values in parentheses are for highest-resolution shell.

a R_sym_ = Σ|I_i_−<I>|/Σ|I_i_| where I_i_ is the intensity of the i^th^ measurement, and <I> is the mean intensity for that reflection.

b R_work_ = Σ| F_obs_−F_calc_|/Σ|F_obs_| where F_calc_ and F_obs_ are the calculated and observed structure factor amplitudes, respectively.

c R_free_ is calculated using the same equation as that for R_work_ but 5% of reflections where chosen randomly and omitted from the refinement.

E2s mainly consists of a single domain that forms a β-barrel. Residues from β2, β3, β6 and β7, along with loops protrude at one side of the β-barrel structure to form a surface groove of approximately 15 Å in width and 11 Å in depth (Figure 1A). The β-barrel consists of nine anti-parallel β-strands running from one end of the molecule to the other. On one side of the β-barrel, there are three loops which connect adjacent β-strands, whereas on the other side, three loops and a double-strand β-sheet connect adjacent β-strands. The pore inside the β-barrel is highly hydrophobic in nature with side chains consisting of thirteen Leu, seven Val, two Ile, three Tyr, two Phe and one Trp, making up a total of 28 hydrophobic residues lining the inner pore surface (Figure S1). Approximate dimensions of the β-barrel are 30 Å in height and 13 Å in diameter. The top and the bottom side of the cavity of the β-barrel are blocked by loops connecting residues Thr586 and Ala590; Ala467 and Phe462 respectively (Figure 1B). Considering the hydrophobic nature and size of the cavity, we postulate that the cavity could have a role in recognizing hydrophobic ligands. However, the exact role of the cavity as yet remains to be established.

### E2s is a dimer

**Figure 2:**
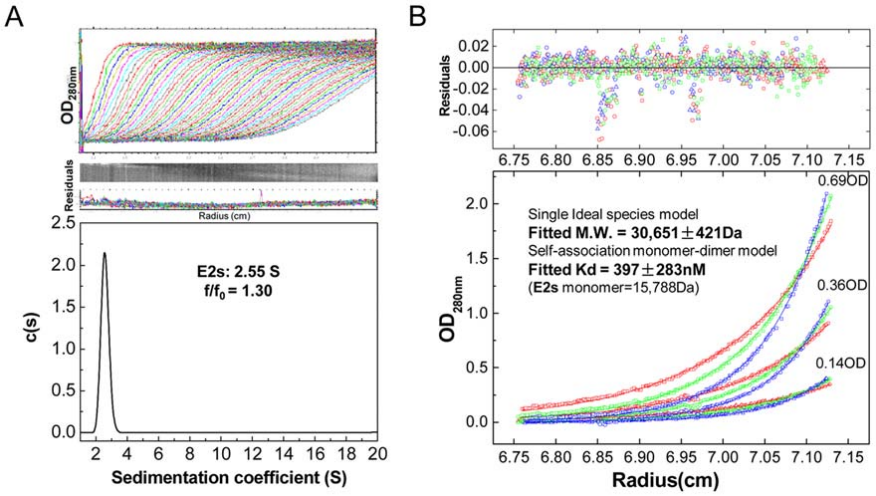
Dimerization of E2s in solution was investigated by analytical ultracentrifugation (AUC). *(A)* Sedimentation velocity experiment shows that E2s behaves as a single globular species having the sedimentation coefficient of 2.55S and a hydrated friction ratio of 1.30. *(B)* Sedimentation equilibrium experiment indicates that E2s mainly exists as a dimer with M.W. 30,651±421 Da. The dissociation constant of the E2s dimer, Kd was fitted as 397±283 nM using the self-association model.

**Figure 3:**
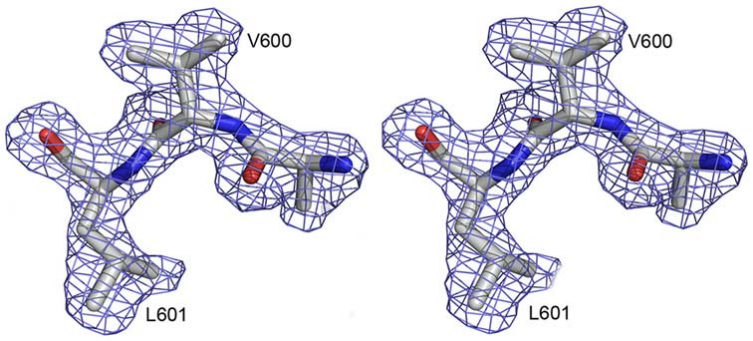
Stereo view of the electron density map. Simulated annealing *Fo-Fc* omit map of the C-terminal region of E2s, which is crucial for the dimerization. Residues Val600, Leu601 and all atoms within 2.0 Å were omitted prior to refinement. The map contoured at a level of 3σ. This figure was prepared using PyMol [34].

E2s was found to exist exclusively as a 2.55 S particle (Figure 2A), which corresponds to a homodimer in solution, with an apparent molecular mass of 30,651±421Da (Figure 2B), as determined by analytical ultracentrifugation (AUC) experiments. These observations were consistent with a dimeric arrangement observed in the crystal structure (symmetry related subunits), with the dimer having approximate dimensions of 54×30×15 Å. The symmetry related subunits of the dimer are packed in a perpendicular fashion to each other, resulting in a maximum interaction (Figure 1C). The strong hydrophobic cluster at the dimer interface is maintained by side chains of residues Val503, Trp548, Thr552, Ala555, Tyr557, Tyr561, Val598 and Val600 of both subunits of the dimer. In addition, eight hydrogen bonding contacts (<3.5 Å) mainly from Arg542, Lys544, Ser546, Thr552, Thr553, Asn562, Thr564 and Ser566 of both subunits are involved in maintaining the dimer architecture. The surface area buried upon dimer formation was calculated using PISA server [10], and was found to be 1142.7 Å^2^, or 16.1% of the total surface of each subunit. The dissociation constant (Kd) was estimated as 397±283 nM by the sedimentation equilibrium method in AUC experiments (Figure 2B). The observed tight dimerization of E2s suggests a functionally important role of the dimer structure. Figure 3 shows the omit map of the C- terminal region which is involved in the dimerization.

### Structural comparison

All the positive-stranded RNA eukaryotic viruses have been shown to have a capsid protein folded as β-barrels with jelly roll topology [11],[12]. These capsid proteins mostly consist of a shell and a projection domain [13],[14]. The shell domain is a tightly closed domain protecting the viral RNA with its jelly roll β-barrel oriented such that the β-strands run tangentially to the particle surface [12]. However, the projection domain, if present, appears to be structurally more variable. The N-terminal region of the HEV capsid protein pORF2 is most likely to represent the shell domain, whereas E2s, the C-terminal region of pORF2, is considered as the projection domain of the HEV capsid protein. A search for topologically similar proteins within the PDB database performed with the program DALI [15] revealed no significant structural homology for E2s. Notably, E2s does not share any significant sequence or structural homology with any known viral proteins. It appears to represent the structurally more variable features of the projection domains.

**Figure 4:**
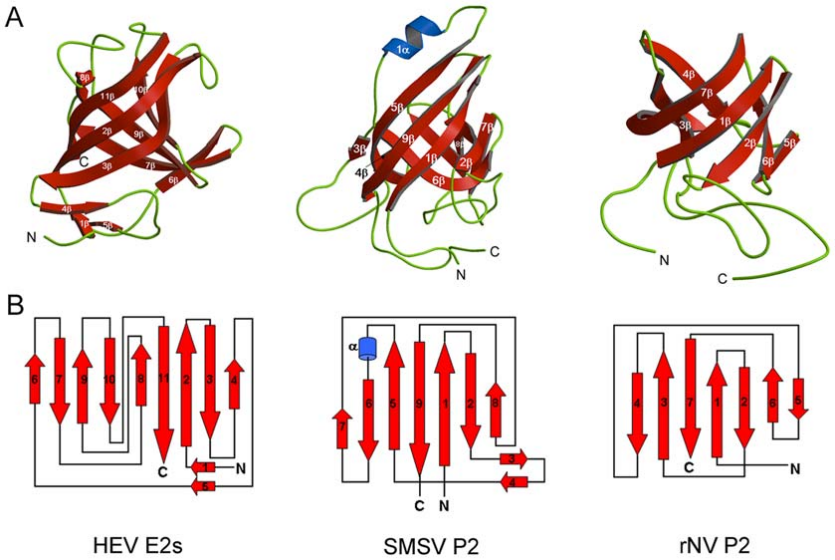
Structural comparison of E2s with P2 domains. *(A)* Side-by-side ribbon diagram of HEV-E2s, the SMSV-P2 domain (pdb code 2gh8) and the rNV-P2 domains (Pdb code 1ihm). β-strands and α-helices are numbered. N- and C-termini are also labeled. These figures were prepared using Molscript and Raster3D [32],[33]. *(B)* Topology diagrams of HEV-E2s, the SMSV-P2 domain and the rNV-P2 domain. β-strands, α-helices and connecting loops are represented by red arrows, blue cylinders and green lines respectively.

It is worth mentioning here that Hepatitis E Virus was initially grouped with *Caliciviruses*, which comprise Norwalk Virus (NV) and San Miguel Sea lion Virus (SMSV). P2 domains of rNV and SMSV have been shown to be recognized by neutralizing monoclonal antibodies, which would suggest that they play a role in virus host interactions [13],[14]. Similar to the E2s domain of HEV, these P2 subdomains of rNV and SMSV are the most exposed regions, and contain determinants of strain specificity for Norwalk Virus (NV) and SMSV, respectively [13],[14]. Therefore, independent structural comparisons of the E2s dimers with the P2 domains of rNV and SMSV were performed. These three domains adopt the β-barrel architecture as shown in Figure 4A and 4B. Because the projection domain of the virus is highly structurally variable, the strand connectivity is not the same between E2s and the other two P2 domain structures (Figure 4B). In addition, the number of β-strands of these three domains is different (Figure 4B). The core part of this domain comprises of nine β-strands in E2s, whereas, there are seven β-strands in both rNV and SMSV P2 subdomain, respectively. However, cores of β-barrels of these three domains can be superposed up to certain extent (RMSD of 3.8 Å for 64 Cα atoms; 7% sequence identity with rNV P2 domain; RMSD of 3.4 Å for 87 Cα atoms; 8% sequence identity with SMSV P2 domain).

### Dimerization of E2 and HEV neutralization

**Figure 5:**
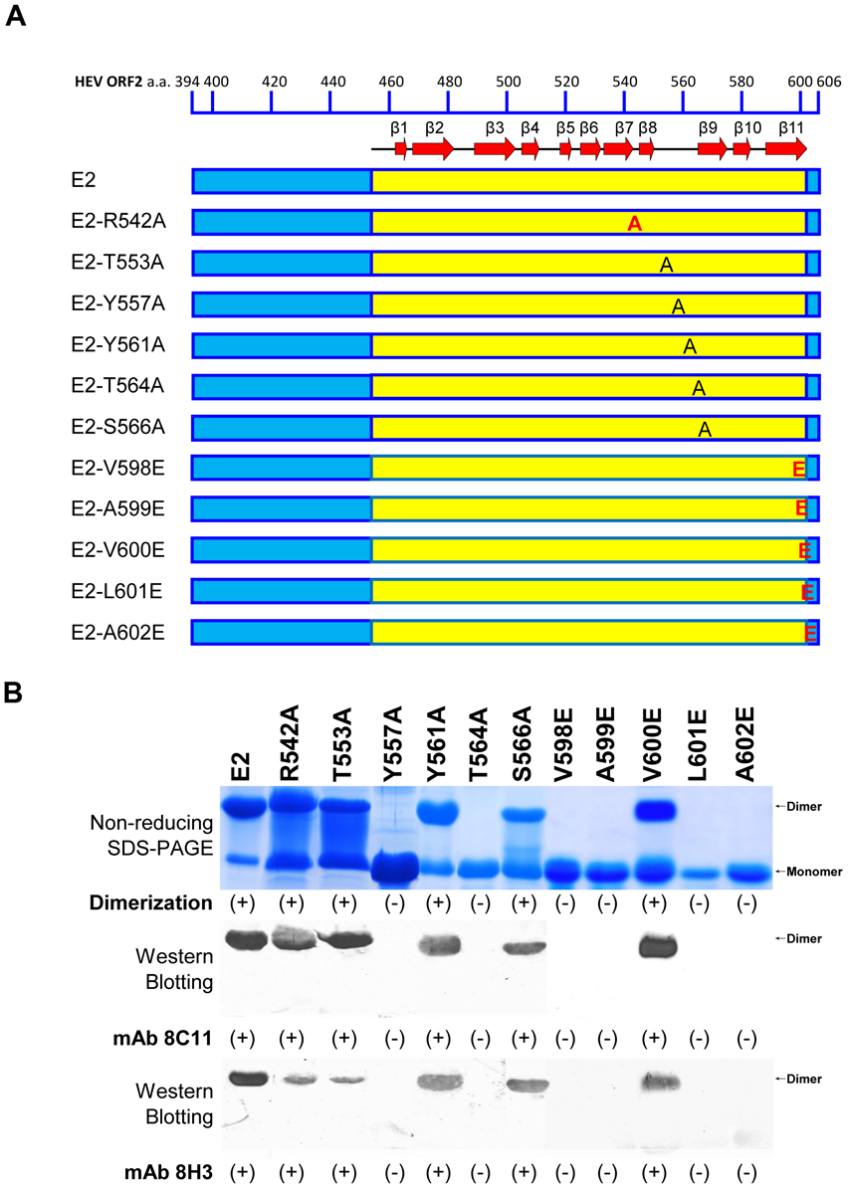
Mutational studies on the dimer interface. *(A)* The schematic representation of wild-type E2 and eleven point mutations targeting the dimer interface region. Secondary structural elements are shown for the E2s region. The mutated residues located on the β-strands and coils are shown in red and black, respectively. *(B)* These mutants and wild-type E2 were subjected to non-reducing SDS-PAGE and Western Blotting with the neutralizing mAb 8C11 and 8H3 to study the effects of these mutations on dimerization and neutralization, respectively. [+] denotes dimerization or reactivity with 8C11 or 8H3, [−] denotes loss of the respective property. Note that both the capacity to form dimers and the reactivity with mAb 8C11 and 8H3 were abolished simultaneously in six of these mutants: Y557A, T564A, V598E, A599E, L601E and A602E.

**Figure 6:**
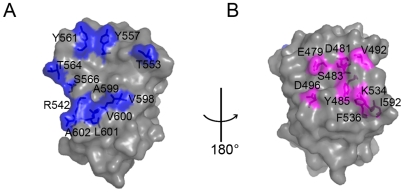
Mapping of site-directed mutation on E2s. A transparent surface representation of the subunit of the E2s dimer is shown at two different orientations. *(A)* Shows the dimerization mutants and *(B)* shows the groove region mutants. Figure 6*(B)* is 180° rotated with respected to Figure 6*(A)*. Further the view of Figure 6*(A)* is related to the view of Figure 1*(A)* with an anticlockwise rotation of 90°. All mutated residues are shown in ball and stick model. Residues playing roles in the E2s dimerization are shown in blue. Residues in the groove region that were mutated to study the reactivity of mAb are shown in magenta. This figure was prepared using PyMol [34].

Analysis of the dimer interface of E2s provided new insight into its tight dimeric architecture. In the case of NV and SMSV the P2 subdomains are dimers and are shown to interact with the host [13],[14],[16],[17],[18]. To verify that the dimerization of E2s is crucial for the host interaction of HEV, several mutations on dimer interface regions of E2/E2s were carried out (Figures 5 and 6), and their roles in destabilizing the dimer formation were studied. A total of eleven point mutants of E2/E2s were constructed. Non-reducing SDS-PAGE was used to verify the dimerization of these mutants in comparison with the standard MW markers (Figure 5). Independently, the dimeric nature of most of E2/E2s constructs was verified by analytical ultracentrifugation and gel filtration experiments (Figures S2 and S3). In addition to C-terminal residues, Arg542 and Tyr557 were revealed by the structure of E2s to be possibly involved in the dimerization. Arg542 is involved in two inter-subunit hydrogen bonding contacts. However, the mutant of Arg542 to Ala was still observed as a dimer in solution and reacted with mAb 8C11. Tyr557 is a part of the hydrophobic cluster, which consists of Tyr559, Tyr561 and Tyr584 from both subunits of the E2s dimer. The mutant of Tyr557 to Ala still showed up as a dimer in solution (Figure S2). However, it migrated as a monomer on a non-reducing SDS-PAGE (Figure 5). It was observed that this mutant retained mAb 8C11 reactivity only as a dimer, but lost its ability to recognize antibodies when it became a monomer in the presence of 0.1% SDS. Furthermore, we observed that five more mutants, namely, E2-T564A, E2-V598E, E2-A599E, E2-L601E and E2-A602E, existed exclusively as monomers in phosphate-buffered saline at pH 7.4. These mutants were found to lack the ability to interact with HEV-neutralizing monoclonal antibody, mAb 8C11 (Figure 5). These results confirm that HEV neutralization sites are associated with the dimeric nature of E2/E2s. In order to further verify the integrity of secondary structures in these mutants, circular dichroism (CD) spectra were recorded for wild-type E2, as well as, for all other mutants. In all cases, CD spectra showed the existence of similar secondary structures (Figure S4).

Moreover, our studies confirmed that various constructs of E2, such as E2a, p239 and E2s, contain the major dimerization site in the C-terminal region between aa597 and aa602 (Table S1) [6]. This is supported by the structure of E2s, which reveals the participation of hydrophobic residues of the β11 strand (Gly589 to Ala602) in forming the dimeric interface.

Previously we had shown that mAb 8C11 and 8H3 neutralize HEV infection in monkeys [8] and block virus-host interaction [7]. Consequently, all E2 constructs were verified for the ability to form dimers and were tested for their reactivity with the neutralizing monoclonal antibodies (Figure 5). The structure of E2s reported here is the representative structure for all E2 constructs. E2s is the shortest among all constructs of E2 (Figure 5) that can dimerize and recognize HEV antibodies, in a way similar to other E2 constructs, as well as the native HEV [8]. Furthermore, E2s contains most of the conserved residues. More importantly, it contains the crucial dimerization region of E2. The structure of E2s shows that C-terminal residues, such as Ala597, Val598, Ala599, Leu601 and Ala602, are directly engaged in dimer formation, with additional interactions from Tyr557 further stabilizing dimers.

### HEV antibody recognition and E2s

We have investigated the functional relevance of E2 constructs through a panel of 33 previously reported mAb that are reactive against the E2 fragment (aa 394–606) and p239 (aa 368–606) (Table S1). These antibodies target the unique structural features of E2/E2s. Thirteen of them are linear epitope - reactive antibodies and 20 of them are conformational determinants. Moreover, two of 13 linear epitope-reactive antibodies and 15 of 20 conformational determinant -reactive antibodies can bind genotype I and/or genotype IV HEV. We have further identified that two of them (mAb 8C11 and 8H3) can neutralize the infectivity of HEV, thus preventing the virus from infecting primates [8]. Notably, one of the recombinant E2 constructs, p239 (ORF2 aa368–606), forms a virus like particle (VLP) with a diameter of 23 nm [9]. The p239 VLP was found to specifically adsorb and penetrate susceptible host cells similarly to live HEV viruses. This interaction could be blocked by the neutralizing mAb 8C11 and 8H3 [7]. This showed that the recombinant E2 constructs shared many common features of the native virus. Thus, it can be suggested that the host and antibody interaction sites/regions of HEV are similar if not identical.

**Figure 7:**
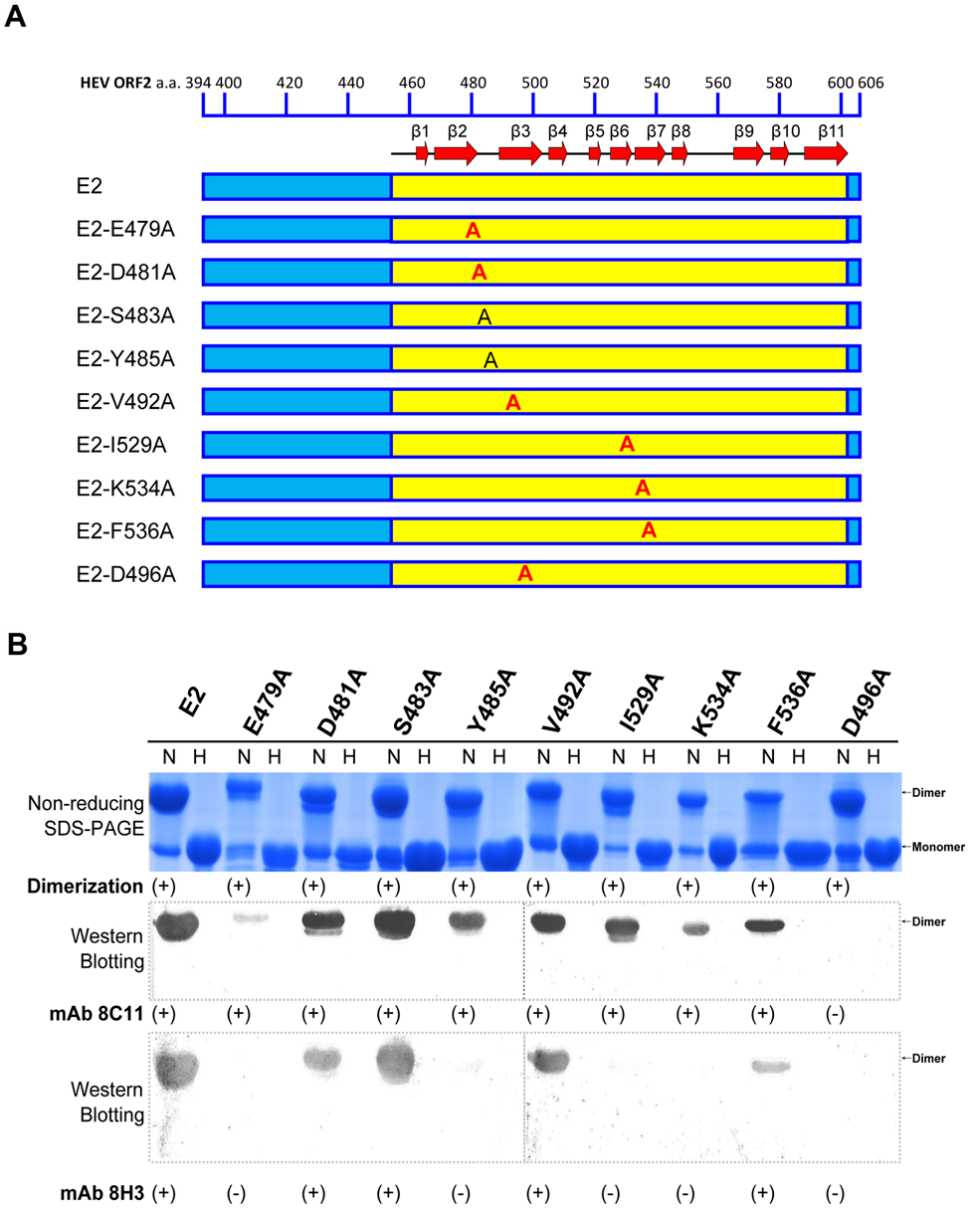
Mutational studies on the groove region. *(A)* The schematic representation of wild-type E2 and nine point mutations targeting the solvent-accessible residues near the groove region. *(B)* The wild type E2 and its mutants were subjected to non-reducing SDS-PAGE and Western Blotting with the HEV-neutralizing antibody 8C11 or 8H3. In this figure the lanes with H indicate samples in the reduced condition (i.e. these samples were heated up to 100°C for 3 minutes). These samples were mainly resolved as monomers. The lanes with N indicate samples in the non-reducing condition (i.e. these samples with 0.1% SDS, no BME and were not heated). These samples were resolved mainly as dimers. All nine mutants remained as dimers. Western Blotting showed that the dimeric E2 wild type and eight mutants were reactive with mAb 8C11. Of these, only E479A, Y485A, I529A, K534A and D496A abolished the 8H3 reactivity. Interestingly, mutant D496A abolished the HEV neutralizing antibodies 8C11 and 8H3 reactivity while maintaining the dimeric arrangement.

Based on our studies on various E2 constructs, we hypothesize that the surface exposed groove region is the most likely antibody recognition site of HEV (Figure 1A). To verify this hypothesis we have carried out several mutations targeting the groove region, and studied their interactions with mAb 8C11 and 8H3 (Figures 6, 7 and S5). Interestingly, the structure of the groove region of E2s is unique, and no such groove region was observed in the P2 domain in either rNV or SMSV [13],[14]. Our analysis showed that (1) all these mutants remained as dimers, (2) only D496A mutant did not recognize mAb 8C11 and 8H3, and (3) mutants E479A, Y485A, I529A and K534A abrogated the reactivity of mAb 8H3, while retaining the 8C11 reactivity (Figure 7B). These mutagenesis studies suggest that the groove region may contain a neutralization site. It is possible that the required positioning of the groove region might be preserved only in the dimeric form of E2, and it might collapse when it becomes a monomer (Figure 7B, lane E2-N, H). Hence we propose that the dimeric form of E2 is essential to position these groove regions for the neutralization of HEV. We see that antibody interactions are associated with the E2 region of HEV, a region which may mediate the first contact with the host cell to initiate viral infection [7]. Thus, the capsid protein domain E2 is a functionally important region of HEV, and it reacts against different mAb that are capable of HEV immune capture and virus neutralization.

## Discussion

HEV is one of the major causes of acute hepatitis in humans and non-human primates. Neutralizing antibodies such as 8C11 and 8H3 bind with native HEV [8], as well as with the dimeric form of E2 constructs [6],[19]. We believe that the interaction sites of these antibodies are located on the surface region of HEV, and that the dimeric E2 domain is, presumably, located in this region of HEV. Furthermore, based on our experimental observations on the mAb recognition of E2 constructs, we strongly believe that the dimeric nature of E2 is functionally relevant. The homodimer E2 domain is the C-terminal part of the major capsid protein of HEV containing the antibody neutralization site of HEV. The present structural and functional studies on the recombinant E2s, a representative construct of E2 domain, demonstrate the important role of dimerization and its implication for virus-host interaction.

Our analysis suggests that the HEV-neutralizing sites, as defined by mAb 8C11 and 8H3, are distinct, because the specificity of each antibody is different. However, mAb reactive sites are strictly associated with the dimeric domain of HEV capsid protein, i.e. the E2 domain. The recognition by these mAb is totally lost with the dissociation of the dimeric form of E2 into its monomeric form. Further mutagenesis of E2 constructs suggests that mAb 8C11 and 8H3 interact with the neutralization sites located near the surface groove region, and that the conformation of the dimeric structure is important for neutralization.

Taken together the presence of neutralizing antibody binding sites of HEV on E2s, and the shape of the dimer, we suggest that E2s is present on the surface protrusions of HEV shown in the electron microscopic structure [5]. The equivalent protruding region of rNV and SMSV is different, and this might be due to structural variations of these protruding domains E2 *vs.* P2 of HEV and rNV/SMSV respectively. These variations may be related to the specific recognition to a specific host cell. Nonetheless, we suggest that the protruding region of the hepatitis E virus is equivalent to the protruding P2 subdomains of the rNV virus and SMSV [13],[14] and both are expected to have a similar role in host interactions. Notably, due to the resemblance in morphology, hepatitis E virus was formerly included in the family *Caliciviridae* (comprising of rNV and SMSV), before it was reclassified to a separate genus, *Hepevirus* in the family *Hepeviridae*, according to the phylogenetic analyses [2],[3]. This protruding region which is likely to harbor the E2s and the antibody binding site of the hepatitis E virus, is crucial for its interaction with the host cell for initiation or propagation of viral infection. This is likely to be a universal mechanism for most virus-host interactions and would warrant further study. Our results can be extended towards vaccine development against HEV infection in humans, and to open up new avenues to design specific inhibitors for the virus.

## Materials and Methods

### Plasmid and strain construction

The E2s gene (encoding HEV ORF2 aa 455–602) was PCR amplified from the E2 gene (ORF2 aa 394–606) [6], then subcloned into a vector pMD 18-T with a TA cloning kit (Takara, Dalian, China), and ligated to a non-fusion pTO-T7 expression plasmid [20] in *Nde*I/*EcoR*I restriction sites. The alanine scanning mutageneses on E2s or E2 were carried out with site-directed PCR reactions. All resultant DNA fragments were cloned into a pTO-T7 plasmid. The *E.coli* ER2566 strain was transformed to express these non-fusion proteins with an additional start Met at N-terminus.

### Purification and crystallization

All recombinant proteins formed inclusion bodies in bacterial cells. Inclusion bodies were separated from cellular debris by extensive washing with buffer containing 2% Triton X-100, and then dissolved by homogenization with 4 M (for E2 and its mutants) or 8 M urea (for E2s and its mutants). Proteins were renatured by dialysis against phosphate-buffered saline at pH 7.4 (137 mM NaCl, 2.7 mM KCl, 10 mM Na_2_HPO_4_ and 2 mM KH_2_PO_4_) at room temperature and further purified by gel filtration HPLC using a TSK gel SW3000 25 mm×60 cm column (TOSOH, Japan). E2s was dialyzed with pure H_2_O and concentrated to 15 mg/ml for crystallization. Crystallization drops containing 1 µl E2s protein solution (15 mg/ml) and 1 µl reservoir solution were equilibrated by hanging drop vapor diffusion at 21°C. The best crystals were grown from a reservoir solution consisting of 0.1 M HEPES pH 7.5, 12% PEG3350 and 5 mM of cobalt chloride hexahydrate, nickel (II) chloride hexahydrate, cadmium chloride dehydrate and magnesium chloride hexahydrate. Crystals measuring ∼0.2 mm in length grew over the course of 3 days, belonged to the space group R32, and contained one molecule in the asymmetric unit. Obtaining the diffraction quality single crystals and the phasing were the most challenging task in this project. The present data set is the best of over one hundred data sets collected. The X-ray data collection and refinement statistics are given in Table 1.

### Data collection, structure solution and refinement

Crystals were cryo-protected in the reservoir solution supplemented with 25–30% glycerol, and flash cooled at 100 K. The structure was determined using Br heavy atom soaked crystals of recombinant E2s protein by the single-wavelength anomalous dispersion (SAD) method. X-ray diffraction data were collected at the beamline X12C, Brookhaven National Laboratory using a Quantum-210 CCD detector (ADSC). A single data-set was collected at the wavelength corresponding to the peak. All data-sets were processed with HKL2000 [21]. Three Br sites of an asymmetric unit were located by using the program BnP [22]. Phases were further improved by density modification using RESOLVE [23],[24], which gave a final overall Figure of merit 0.70. Over 67% of the backbone atoms of the model were built by the RESOLVE iteration method [25]. Remaining residues of the molecules were added after several cycles of manual model building by using O [26], followed by refinement using CNS [27]. Finally, 244 well-defined water molecules were added, and refinement was continued until the R-value converged to 0.198 (R_free_ = 0.240) for reflections I>σ (I) to 2.0 Å resolution. The model had good stereochemistry, with all residues within allowed regions of the Ramachandran plot (Table 1) analyzed by PROCHECK [28].

### Analytical Ultra-Centrifugation (AUC)

The AUC velocity experiment was to independently establish the homogeneity of the molecules in solution, and subsequently determine their molecular mass by equilibration experiment. Sedimentation velocity (SV) and sedimentation equilibrium (SE) experiments were conducted at 20°C on a Beckman XL-A analytical ultracentrifuge, equipped with absorbance optics and an An60-Ti rotor. The molecular mass and partial specific volume of E2s, the solvent density and viscosity of the solvent were calculated from the amino acid or buffer composition using the program SEDNTERP (John Philo, Amgen, Thousand Oaks, CA, and RASMB). For SV experiments E2s was diluted to 1 mg/ml (∼1.2 OD_280 nm_) in phosphate-buffered saline at pH 7.4. The rotor speed was set at 60,000 rpm for the highest resolution. The sedimentation coefficient and f/f_0_ were obtained with c(s) method [29] using the Sedfit software (kindly provided by Dr. P. Schuck, National Institutes of Health, http://www.analyticalultracentrifugation.com). Similarly, for SE experiments E2s was diluted to 0.69OD, 0.36OD and 0.14OD in phosphate-buffered saline at pH 7.4 (137 mM NaCl, 2.7 mM KCl, 10 mM Na_2_HPO_4_ and 2 mM KH_2_PO_4_). Samples were centrifuged, first at 18,000 rpm, and, subsequently, at 21000, 24000 rpm, respectively, and finally at 42000 rpm for solute depletion. Data sets were processed using the program Origin (Beckman) for detecting multiple equilibria and were fitted to a single ideal species model and a self-association monomer-dimer model using a nonlinear least squares fit [30]. In subsequent models the monomer molecular mass was fixed at the value calculated from the E2s amino acid sequence (15,788 Da).

### SDS-PAGE and Western Blotting (WB)

Analysis of proteins by SDS-PAGE was performed according to the method of Laemmli with minor modifications [31]. Polyacrylamide gels with 12% or 15% acrylamide in the separating gel and 5% in the stacking gel were used. Protein samples were mixed with equal volumes of 2× loading buffer (100 mmol/L Tris-HCl pH 6.8, 200 mmol/L BME, 4% SDS, 0.2% Bromophenol blue and 20% Glycerol). Sample mixtures were heated at 100°C for 3 minutes and subsequently loaded onto the separating gel. For the non-reducing SDS gel, the buffer contained only 0.1% SDS, no BME, and the sample was not boiled.

For Western Blotting experiments, separated proteins were transferred from an SDS gel onto a nitrocellulose membrane. Membranes were soaked in 1∶2,000 diluted HEV-neutralizing monoclonal antibody (8C11 or 8H3), incubated at room temperature for 1 h, and subsequently washed with 0.2% Tween 20 in phosphate-buffered saline (at pH 7.4). The bound antibody was detected with alkaline phosphatase conjugated secondary antibody (DAKO), and developed with a mixture of nitro blue tetrazolium and 5-bromo-4-chloro-3-indolyl phosphate.

### Gel filtration chromatography

Purified proteins were passed through a Superdex 75 10/300GL column equilibrated in phosphate-buffered saline (at pH 7.4) using an AKTA Explorer 100 (GE, USA) at a flow rate of 0.5 ml/min. Molecular weights of eluted proteins were determined by using the following molecular weight standards: Conalbumin (75 kDa), Ovalbumin (43 kDa), Carbonic Anhydrase (29 kDa), Ribonuclease A (13.7 kDa), and Aprotinin (6.5 kDa) (GE, USA).

### Circular dichroism spectrometry

Far UV spectra (260–190 nm) of the E2 wild-type and mutants were measured in a phosphate-buffered saline at pH 7.4 at room temperature using a J-810 spectropolarimeter (JASCO, Tokyo), with 0.1 cm path length and stoppered cuvettes. A total of 5 scans were recorded and averaged for each spectrum, and the baseline was subtracted.

### Coordinates

Coordinates have been deposited in the Protein Data Bank (accession code 3GGQ).

## Supporting Information

Table S1 Reactivity of E2, p239 and E2s against a panel of 33 mAbs.
(0.07 MB DOC)

Figure S1 The Cα trace of the HEV E2s β-barrel shown in green, top view. The hydrophobic side chains of the residues from the cavity region are shown in thick lines. This figure was prepared by using Molscript and Raster3D [31],[32].
(0.29 MB TIF)

Figure S2 The dimerization of E2s-Y557A in solution was investigated by sedimentation equilibrium experiment in analytical ultracentrifugation (AUC). The results indicate that E2s-Y557A mainly exists as a dimer with M.W. 29,420±97 Da.
(1.68 MB TIF)

Figure S3 Gel filtration chromatography of E2s. By comparison with the molecular weight standards [Conalbumin (75 kDa), Ovalbumin (43 kDa), Carbonic Anhydrase (29 kDa), Ribonuclease A ovalbumin (13.7 kDa), and Aprotinin (6.5 kDa)], the apparent molecular weight of E2s elution fraction was estimated to be 28.5 kDa which corresponds to the molecular weight of the dimeric form.
(0.34 MB TIF)

Figure S4 Circular dichroism (CD) spectra of E2 and its mutants. Curve 1 in red: E2 wild-type. Curve 2 in green: E2-T564A, which becomes a monomer in solution and abolishes the reactivity with the HEV-neutralizing antibodies 8C11 and 8H3. Curve 3 in blue: E2-D496A, which has a mutation near the groove region, and which abolishes the reactivity with HEV-neutralizing antibodies 8C11 and 8H3, but remains a dimer in solution. These CD spectra show that all three viriants have similar β-sheet secondary structures, with peaks at 203 nm, 225 nm and troughs at 199 nm, 209 nm and 229 nm.
(0.15 MB TIF)

Figure S5 SDS-PAGE analysis of E2 and its mutant, D496A. Lane M is the marker. Lane 1 and 3 are samples in the presence of 0.1% SDS (non reduced condition). Lane 2 and 4: Samples were heated at 100°C for 3 minutes with SDS and BME. Apparent molecular weight was estimated by comparing with the molecular weight markers (M). The wild-type E2 is in lanes 1 and 2, whereas E2-D496A is in lanes 3 and 4.
(1.49 MB TIF)
