# Supplementary material for: Dimerization of Hepatitis E Virus Capsid Protein E2s Domain Is Essential for Virus–Host Interaction
Source: PLoS Pathog. 2009 Aug 7;5(8):e1000537. doi: 10.1371/journal.ppat.1000537 (PMC2714988; doi:10.1371/journal.ppat.1000537)
Supplement: Table S1 — Reactivity of E2, p239 and E2s against a panel of 33 mAbs. (0.07 MB DOC) [file ppat.1000537.s001.doc]

**Table S1** Reactivity of E2, p239 and E2s against a 33 mAb panel

| mAb | Antigenic determinants | Immune capture of HEV type I | Immune capture of HEV type IV | E2  (aa394-606) | p239  (aa368-606) | E2s  (aa455-602) |
| --- | --- | --- | --- | --- | --- | --- |
| 8C11* | C | + | - | + | + | + |
| 13D8 | C | + | n/d | + | + | + |
| 9F7 | C | + | + | + | + | + |
| 12F12 | C | + | + | + | + | + |
| 8E10 | C | + | + | + | + | + |
| 12A7 | C | + | + | + | + | + |
| 12G8 | C | + | + | + | + | + |
| 1E8 | C | + | n/d | + | + | + |
| 1A5 | C | + | - | + | + | + |
| 3G3 | C | + | - | + | + | + |
| 8D2 | C | - | - | + | + | + |
| 11E12 | C | - | - | + | + | + |
| 9B2 | C | n/d | n/d | + | + | + |
| 3A11 | L | n/d | n/d | + | + | + |
| 8E6 | L | - | - | + | + | + |
| 8G12 | C | + | + | + | + | - |
| 1D5 | C | + | + | + | + | - |
| 4C10 | C | + | + | + | + | - |
| 8H3* | C | + | + | + | + | - |
| 3B8 | C | + | - | + | + | - |
| 6F8 | C | n/d | - | + | + | - |
| 6E6 | C | n/d | n/d | + | + | - |
| 13G9 | L | + | + | + | + | - |
| 12A10 | L | + | - | + | + | - |
| 11E8 | L | - | + | + | + | - |
| 1B7 | L | - | - | + | + | - |
| 9H7 | L | - | - | + | + | - |
| 6E9 | L | - | - | + | + | - |
| 3G5 | L | - | - | + | + | - |
| 3G4 | L | - | - | + | + | - |
| 4A6 | L | - | n/d | + | + | - |
| 15B2 | L | n/d | n/d | + | + | - |
| 16D7 | L | n/d | n/d | + | + | - |

* the mAb (monoclonal antibody) can neutralize HEV type I in primate infection experiment [8]; C, conformational determinant; L, linear epitope; n/d, not done.
