# Supplementary figures and images for: Dimerization of Hepatitis E Virus Capsid Protein E2s Domain Is Essential for Virus–Host Interaction
Source: PLoS Pathog. 2009 Aug 7;5(8):e1000537. doi: 10.1371/journal.ppat.1000537 (PMC2714988; doi:10.1371/journal.ppat.1000537)

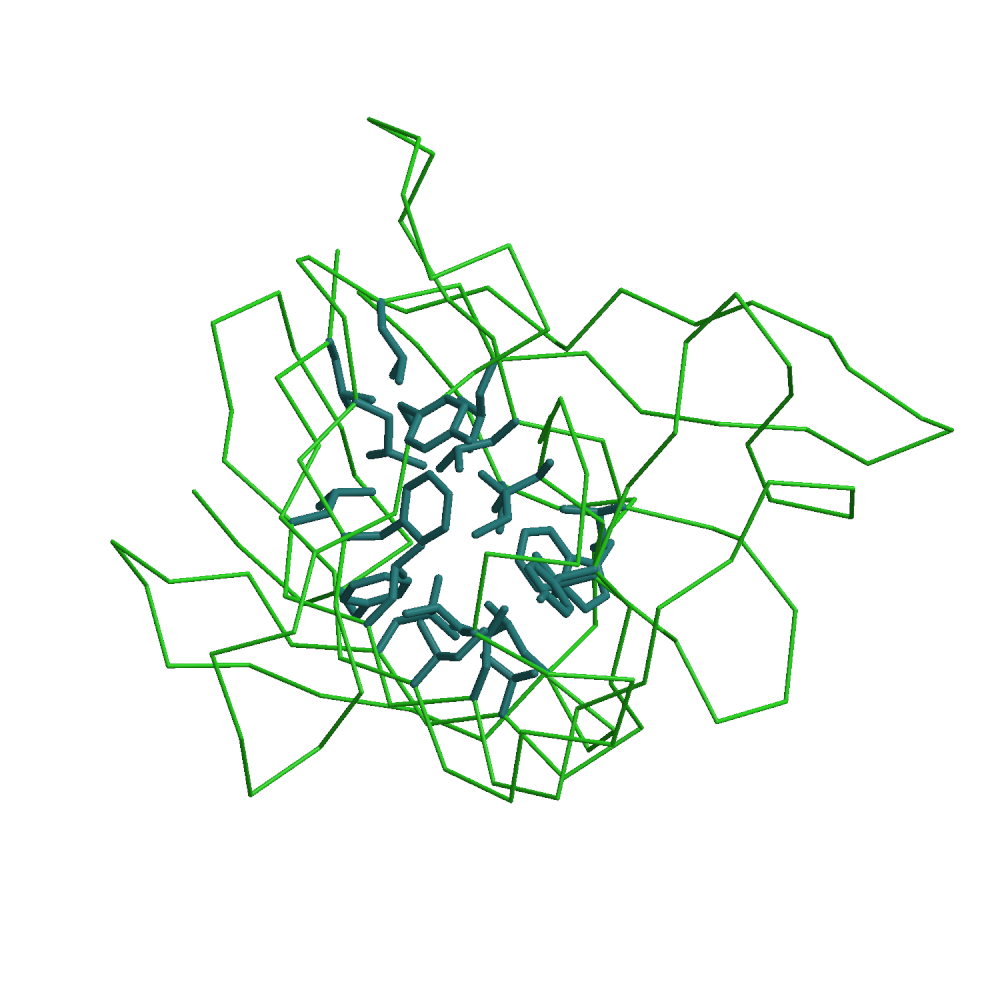

Supplement: Figure S1 — The Cα trace of the HEV E2s β-barrel shown in green, top view. The hydrophobic side chains of the residues from the cavity region are shown in thick lines. This figure was prepared by using Molscript and Raster3D [31],[32]. (0.29 MB TIF) [file ppat.1000537.s002.tif]

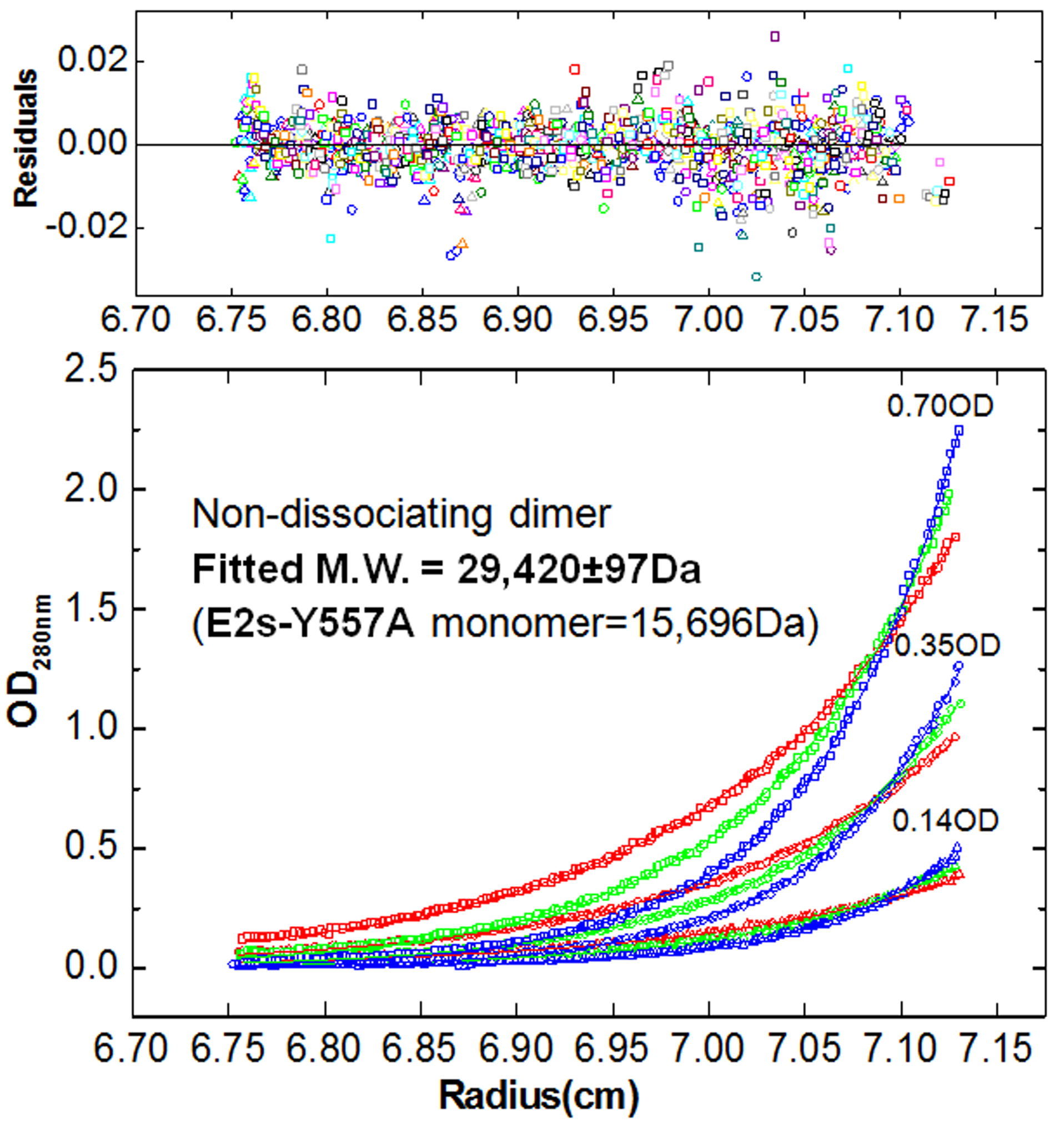

Supplement: Figure S2 — The dimerization of E2s-Y557A in solution was investigated by sedimentation equilibrium experiment in analytical ultracentrifugation (AUC). The results indicate that E2s-Y557A mainly exists as a dimer with M.W. 29,420±97 Da. (1.68 MB TIF) [file ppat.1000537.s003.tif]

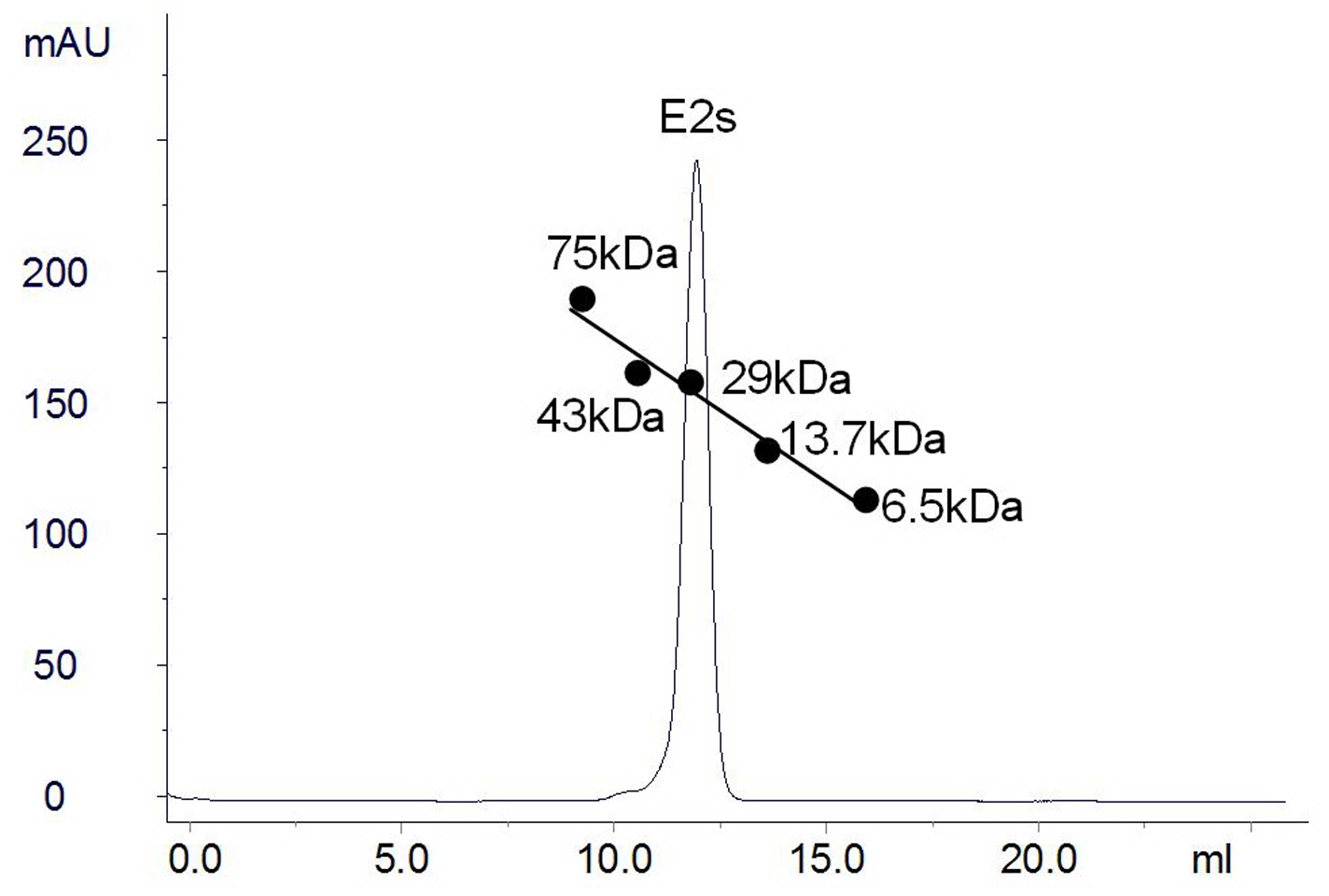

Supplement: Figure S3 — Gel filtration chromatography of E2s. By comparison with the molecular weight standards [Conalbumin (75 kDa), Ovalbumin (43 kDa), Carbonic Anhydrase (29 kDa), Ribonuclease A ovalbumin (13.7 kDa), and Aprotinin (6.5 kDa)], the apparent molecular weight of E2s elution fraction was estimated to be 28.5 kDa which corresponds to the molecular weight of the dimeric form. (0.34 MB TIF) [file ppat.1000537.s004.tif]

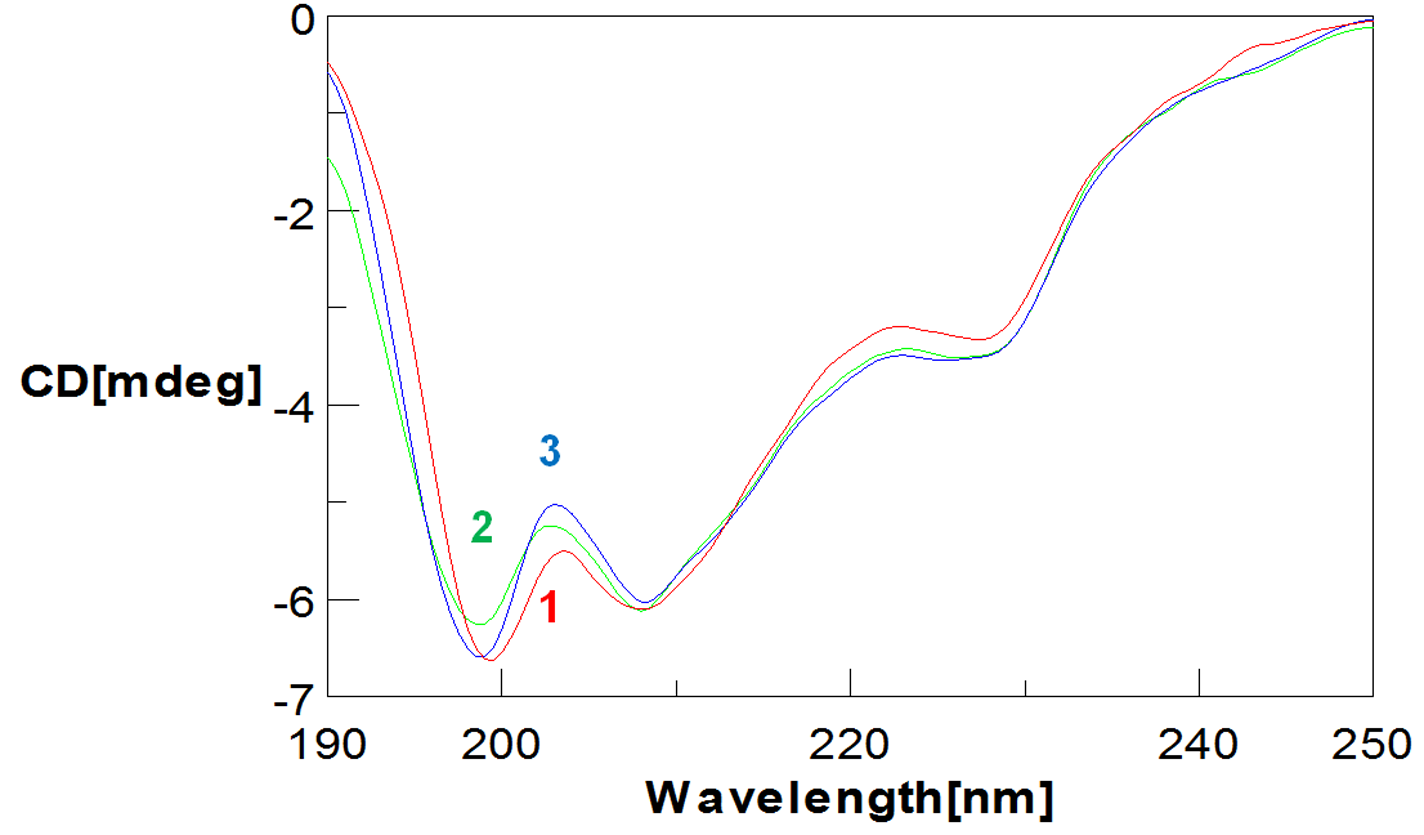

Supplement: Figure S4 — Circular dichroism (CD) spectra of E2 and its mutants. Curve 1 in red: E2 wild-type. Curve 2 in green: E2-T564A, which becomes a monomer in solution and abolishes the reactivity with the HEV-neutralizing antibodies 8C11 and 8H3. Curve 3 in blue: E2-D496A, which has a mutation near the groove region, and which abolishes the reactivity with HEV-neutralizing antibodies 8C11 and 8H3, but remains a dimer in solution. These CD spectra show that all three viriants have similar β-sheet secondary structures, with peaks at 203 nm, 225 nm and troughs at 199 nm, 209 nm and 229 nm. (0.15 MB TIF) [file ppat.1000537.s005.tif]

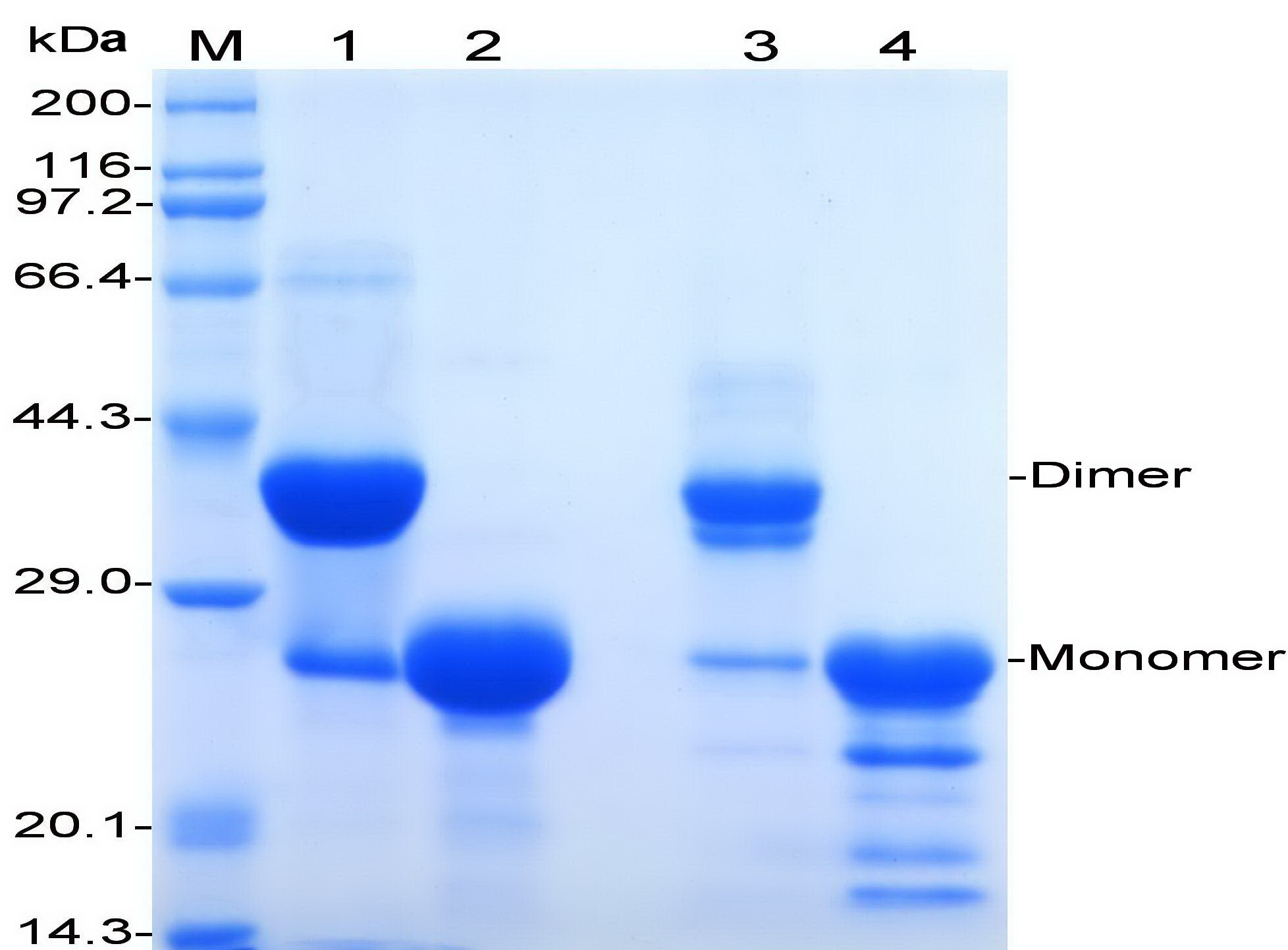

Supplement: Figure S5 — SDS-PAGE analysis of E2 and its mutant, D496A. Lane M is the marker. Lane 1 and 3 are samples in the presence of 0.1% SDS (non reduced condition). Lane 2 and 4: Samples were heated at 100°C for 3 minutes with SDS and BME. Apparent molecular weight was estimated by comparing with the molecular weight markers (M). The wild-type E2 is in lanes 1 and 2, whereas E2-D496A is in lanes 3 and 4. (1.49 MB TIF) [file ppat.1000537.s006.tif]
